# Supplementary figures and images for: The helper NLR immune protein NRC3 mediates the hypersensitive cell death caused by the cell-surface receptor Cf-4
Source: PLoS Genet. 2022 Sep 22;18(9):e1010414. doi: 10.1371/journal.pgen.1010414 (PMC9543701; doi:10.1371/journal.pgen.1010414)

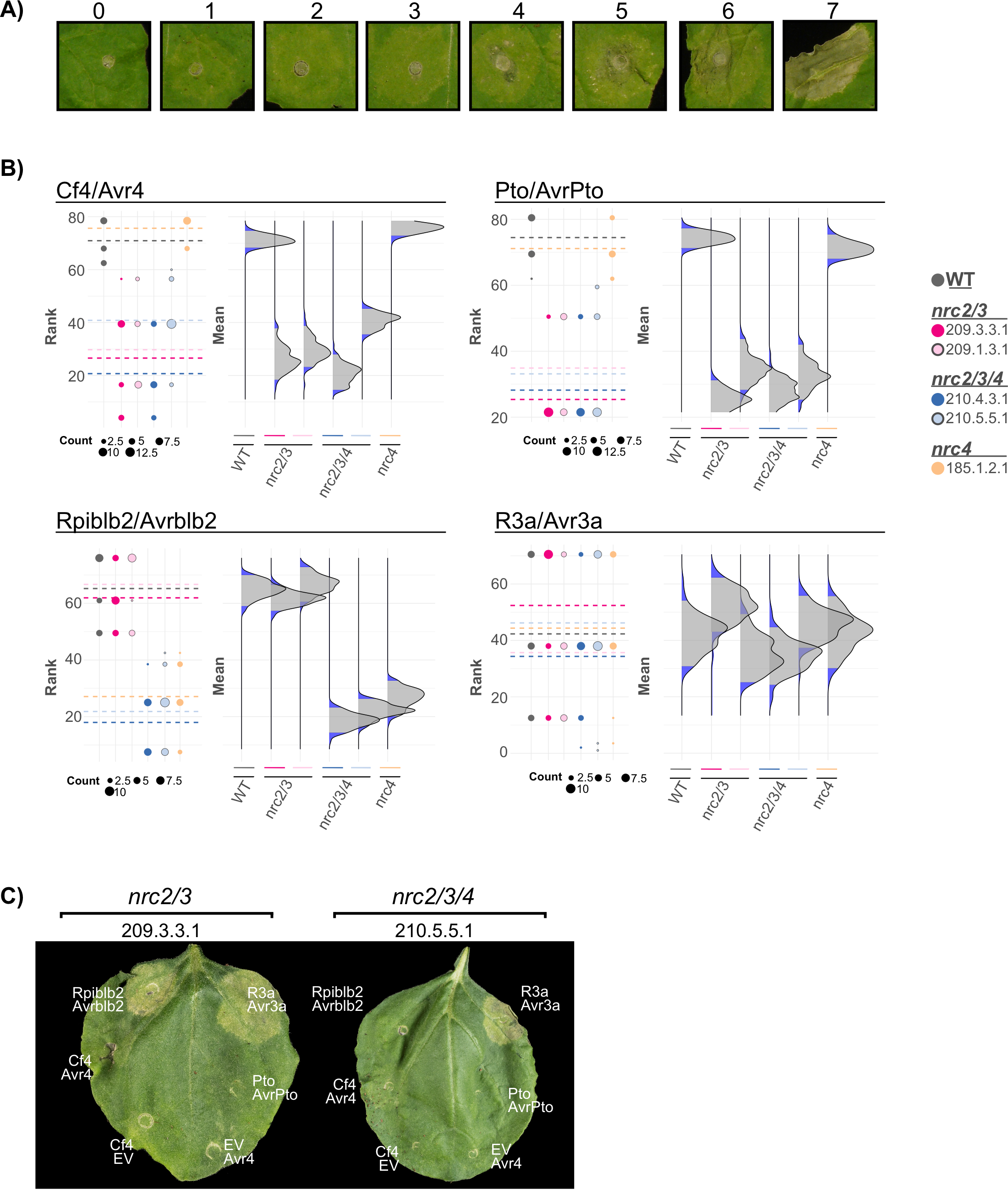

Supplement: S1 Fig — A) Cf-4/Avr4-triggered cell death was scored on a scale of 0–7, with 0 being no response, and 7 being fully confluent cell-death in the entire infiltrated sector. Visible cell death starts appearing at a score of 4. B) Statistical analysis was conducted using the besthr R package. The dots represent the ranked data and their corresponding means (dashed lines), with the size of each dot proportional to the number of observations for each specific value (count key below each panel). The panels on the right show the distribution of 100 bootstrap sample rank means, where the blue areas under the curve illustrate the 0.025 and 0.975 percentiles of the distribution. A difference is considered significant if the ranked mean for a given condition falls within or beyond the blue percentile of the mean distribution of the wild-type control. C) Cf-4/Avr4-triggered hypersensitive cell death is lost in the nrc2/3 and nrc2/3/4 CRISPR lines. Representative N. benthamiana leaves infiltrated with appropriate constructs were photographed 7–10 days after infiltration. The NRC CRISPR lines, nrc2/3-209.3.3.1, nrc2/3/4-210.5.5.1, are labelled above the leaf and the receptor/effector pair tested, Cf-4/Avr4, Prf (Pto/AvrPto), Rpi-blb2/AVRblb2 or R3a/Avr3a, are labelled on the leaf image. Cf-4/EV and EV/Avr4 were also included. (TIF) [file pgen.1010414.s001.tif]

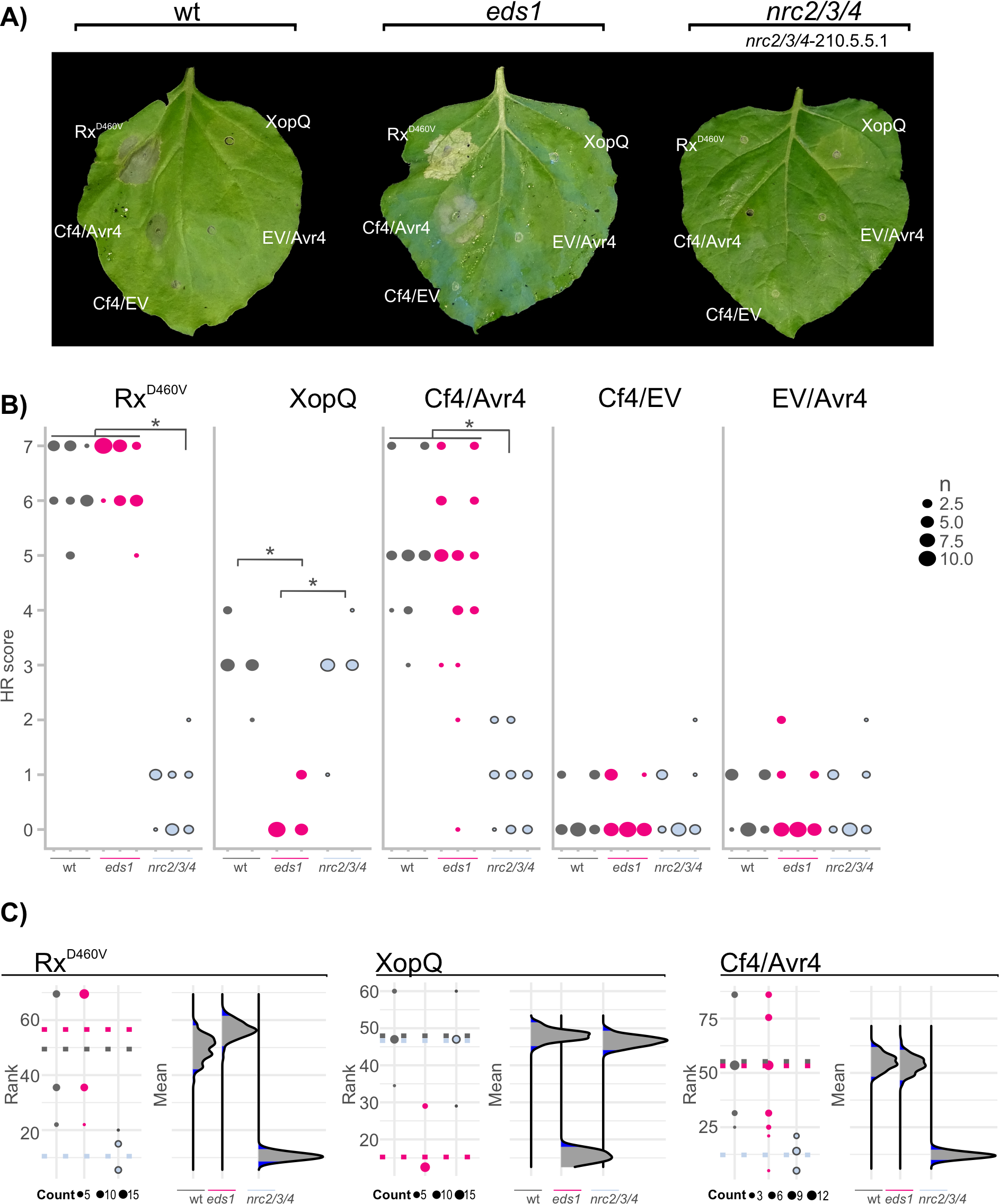

Supplement: S2 Fig — A) Cf-4/Avr4-triggered hypersensitive cell death is lost in the nrc2/3/4 knock-out lines, but not in the eds1 line. Representative N. benthamiana leaves infiltrated with appropriate constructs photographed 7–10 days after infiltration. The CRISPR lines, nrc2/3/4-210.4.3.1, and eds1, are labelled above the leaf and the receptor/effector pair tested, Cf-4/Avr4 (NRC2/3-depedent), Rx/CP (NRC2/3/4-dependent) or XopQ (Roq1, EDS1-dependent), are labelled on the leaf image. As noticed previously, transient XopQ expression gives a mild chlorotic response which is absent in the eds1 CRISPR line [70]. Cf-4/EV and EV/Avr4 were also included as negative controls. B) Quantification of hypersensitive cell death. Cell death was scored based on a 0–7 scale (S1 Fig) between 7–10 days post infiltration. The results are presented as a dot plot, where the size of each dot is proportional to the count of the number of samples with the same score within each biological replicate. The experiment was independently repeated three times. The columns correspond to the different biological replicates. Significant differences between the conditions are indicated with an asterisk (*). C) Statistical analysis was conducted using the besthr R package. The dots represent the ranked data and their corresponding means (dashed lines), with the size of each dot proportional to the number of observations for each specific value (count key below each panel). The panels on the right show the distribution of 100 bootstrap sample rank means, where the blue areas under the curve illustrate the 0.025 and 0.975 percentiles of the distribution. A difference is considered significant if the ranked mean for a given condition falls within or beyond the blue percentile of the mean distribution of the wild-type control. (TIF) [file pgen.1010414.s002.tif]

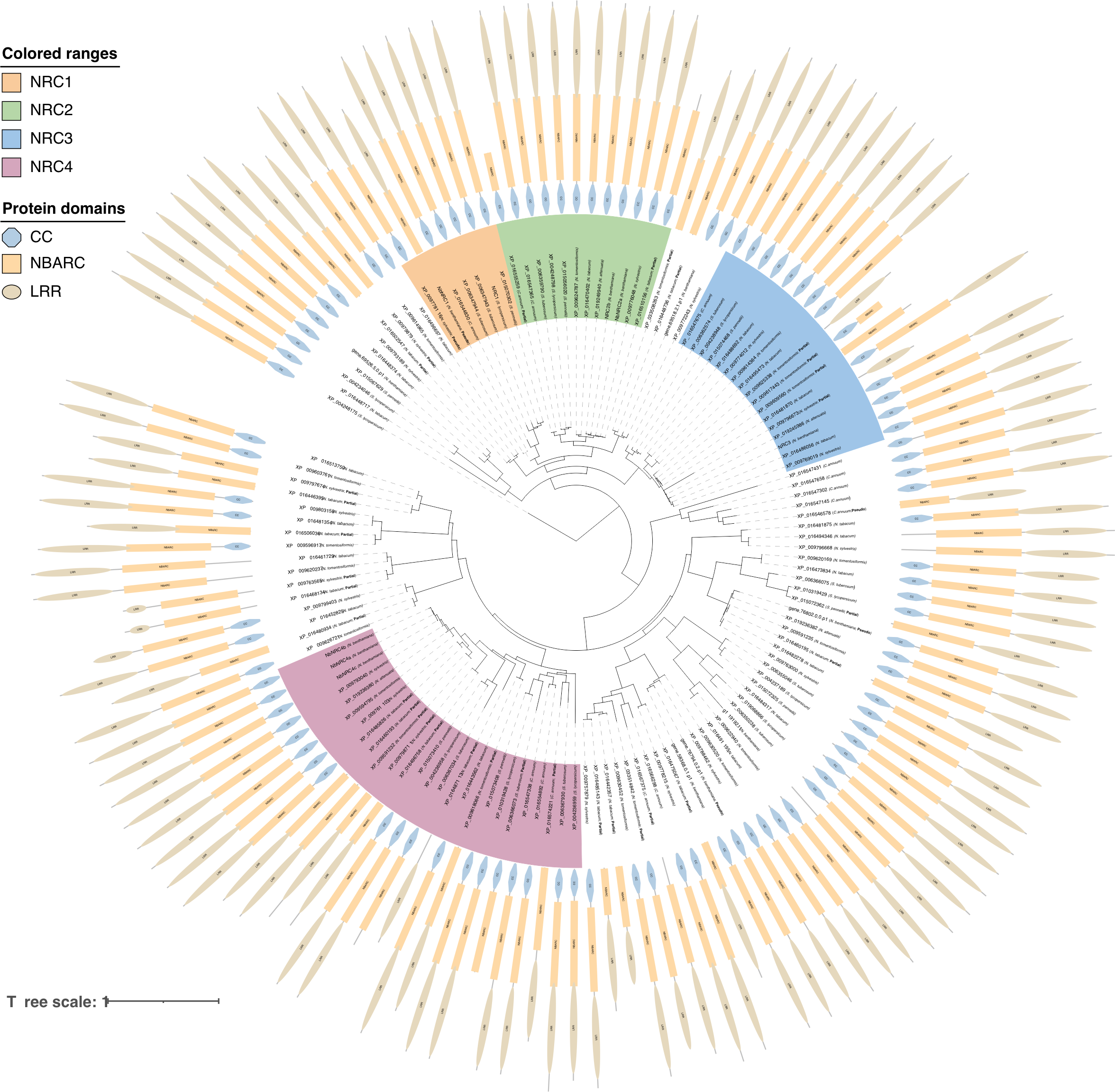

Supplement: S3 Fig — Phylogenetic tree of the NRC-helper NLR family based on the full-length protein sequences was inferred using an approximately Maximum Likelihood method as implemented in FastTree [68] based on the Jones-Taylor-Thornton (JTT) model [71]. The tree was rooted on XP_00428175. The different NRC subfamilies are indicated. Domain architecture was determined using NLRtracker [3]. Probable pseudogenes or partial genes, including Nicotiana NRC1 are indicated. (TIF) [file pgen.1010414.s003.tif]

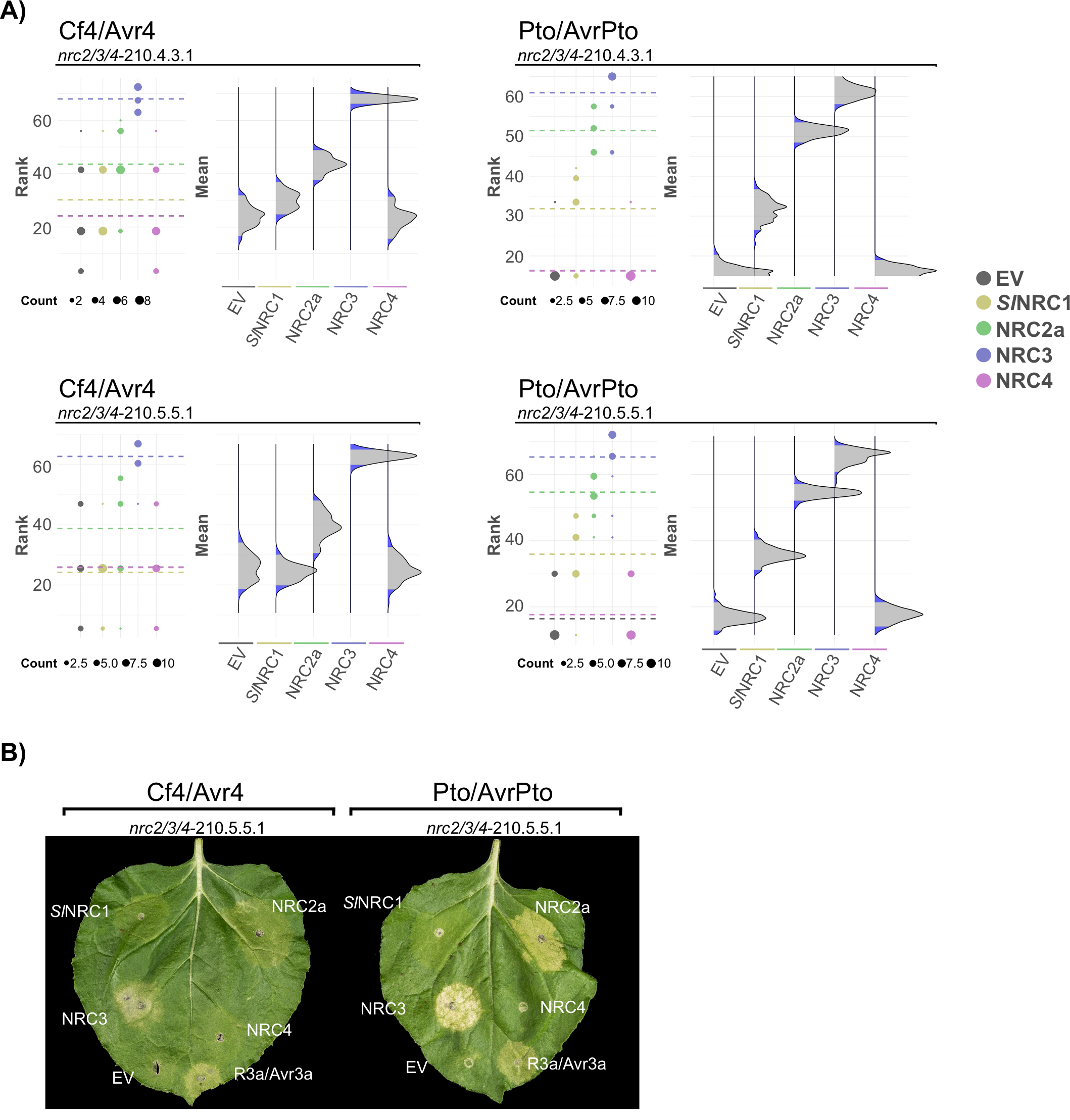

Supplement: S4 Fig — A) Statistical analysis was conducted using besthr R package [72]. The dots represent the ranked data and their corresponding means (dashed lines), with the size of each dot proportional to the number of observations for each specific value (count key below each panel). The panels on the right show the distribution of 100 bootstrap sample rank means, where the blue areas under the curve illustrate the 0.025 and 0.975 percentiles of the distribution. A difference is considered significant if the ranked mean for a given condition falls within or beyond the blue percentile of the mean distribution of the wild-type control. B) Cf-4/Avr4-triggered hypersensitive cell death is complemented in the nrc2/3/4 CRISPR lines with NRC3 and to a lesser extent NRC2, but not NRC1 or NRC4. Representative N. benthamiana leaves infiltrated with appropriate constructs were photographed 7–10 days after infiltration. The receptor/effector pair tested, Cf-4/Avr4 and Prf (Pto/AvrPto), are labelled above the leaf of NRC CRISPR line nrc2/3/4-210.5.5.1. The NRC used for complementation or EV control are labelled on the leaf image. (TIF) [file pgen.1010414.s004.tif]

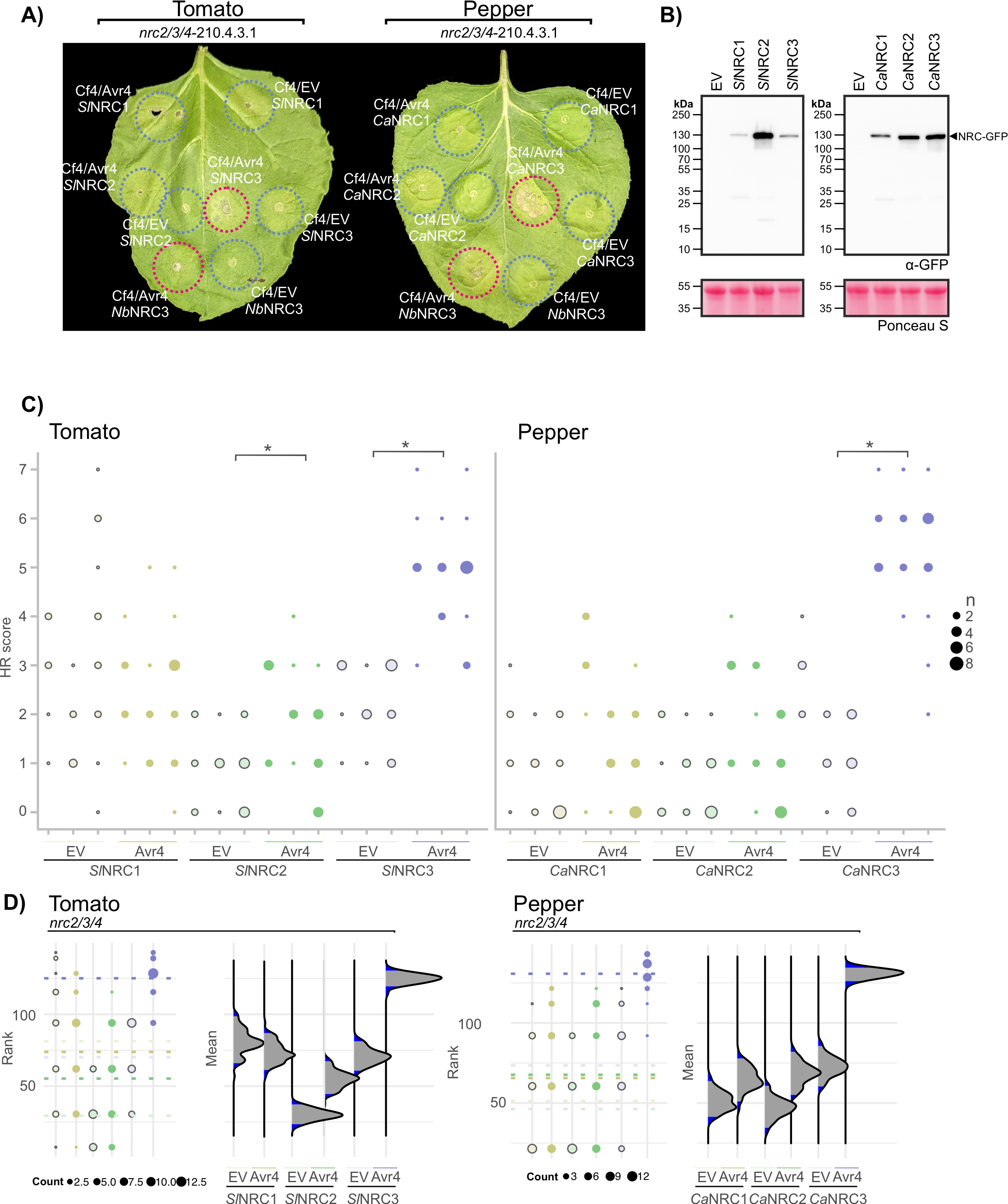

Supplement: S5 Fig — A) Cf-4/Avr4-triggered hypersensitive cell death is complemented in the nrc2/3/4 lines with tomato and pepper NRC3 (SlNRC3 and CaNRC3), but not tomato or pepper NRC1 (SlNRC1 and CaNRC1), or NRC2 (SlNRC2 and CaNRC2). Representative N. benthamiana leaves infiltrated with appropriate constructs were photographed 7–10 days after agroinfiltration. The receptor/effector pair tested, Cf-4/Avr4 or Cf-4/Avr2 as a negative control, and the NRC used for complementation are labelled on the leaf image. Hypersensitive cell-death is indicated by magenta circles, whereas combinations not displaying hypersensitive cell-death are marked with cyan circles. B) SlNRC1, SlNRC2, and SlNRC3 and CaNRC1, CaNRC2, and CaNRC3 protein accumulation. Immunoblot analysis of transiently expressed C-terminally mEGFP-tagged NRCs together with the p19 silencing inhibitor 5 days after agroinfiltration in wild-type N. benthamiana plants detected using anti-GFP antibody. P19 alone was used as an empty vector control. C) Quantification of hypersensitive cell death. Cell death was scored based on a 0–7 scale between 7–10 days post infiltration. The results are presented as a dot plot, where the size of each dot is proportional to the count of the number of samples with the same score within each biological replicate. The experiment was independently repeated three times. The columns correspond to the different biological replicates. Significant differences between the conditions are indicated with an asterisk (*). Some of the codon-optimized NRC constructs display weak autoactivity. D) Statistical analysis was conducted using besthr R package [72]. The dots represent the ranked data and their corresponding means (dashed lines), with the size of each dot proportional to the number of observations for each specific value (count key below each panel). The panels on the right show the distribution of 100 bootstrap sample rank means, where the blue areas under the curve illustrate the 0.025 and 0.975 percentiles [file pgen.1010414.s005.tif]

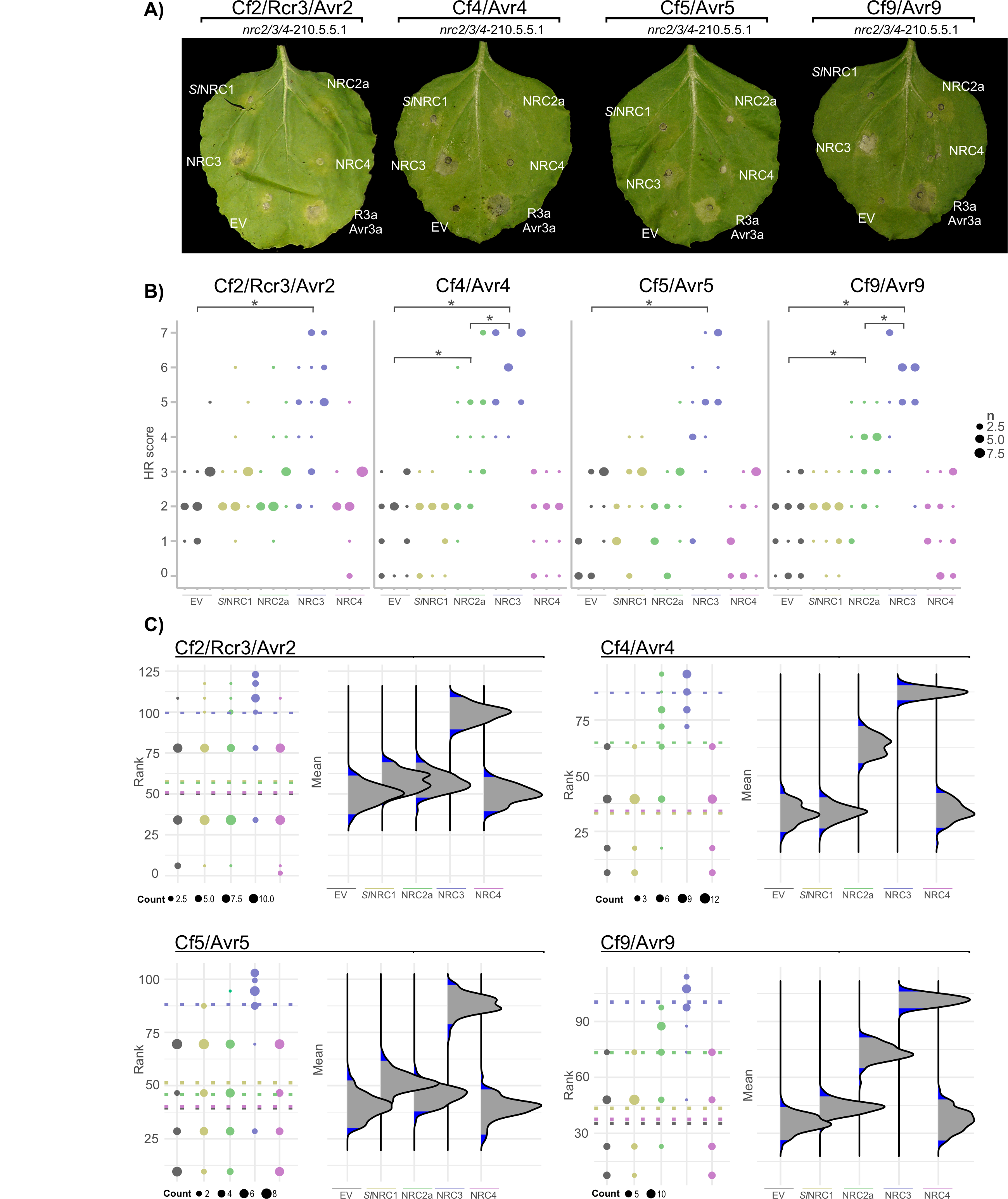

Supplement: S6 Fig — A) Cf-2/Rcr3/Avr2-, Cf-5/Avr5-, and Cf-9/Avr9-triggered hypersensitive cell death is complemented in the nrc2/3/4 lines with NRC3, while Cf-9/Avr9-triggered hypersensitive cell death is partially complemented with NRC2. Representative N. benthamiana leaves infiltrated with appropriate constructs were photographed 7–10 days after agroinfiltration. The receptor/effector pair tested, Cf-2/Rcr3/Avr2, Cf-5/Avr5, and Cf-9/Avr9, are labelled above the leaf of nrc2/3/4-210.4.3.1. The NRC used for complementation or EV control are labelled on the leaf image, R3a/Avr3a was used as a positive control. B) Quantification of hypersensitive cell death. Cell death was scored based on a 0–7 scale between 7–10 days post infiltration. The results are presented as a dot plot, where the size of each dot is proportional to the count of the number of samples with the same score within each biological replicate. The experiment was independently repeated three times. The columns correspond to the different biological replicates. Significant differences between the conditions are indicated with an asterisk (*). C) Statistical analysis was conducted using besthr R package [72]. The dots represent the ranked data and their corresponding means (dashed lines), with the size of each dot proportional to the number of observations for each specific value (count key below each panel). The panels on the right show the distribution of 100 bootstrap sample rank means, where the blue areas under the curve illustrate the 0.025 and 0.975 percentiles of the distribution. A difference is considered significant if the ranked mean for a given condition falls within or beyond the blue percentile of the mean distribution of the wild-type control. (TIF) [file pgen.1010414.s006.tif]

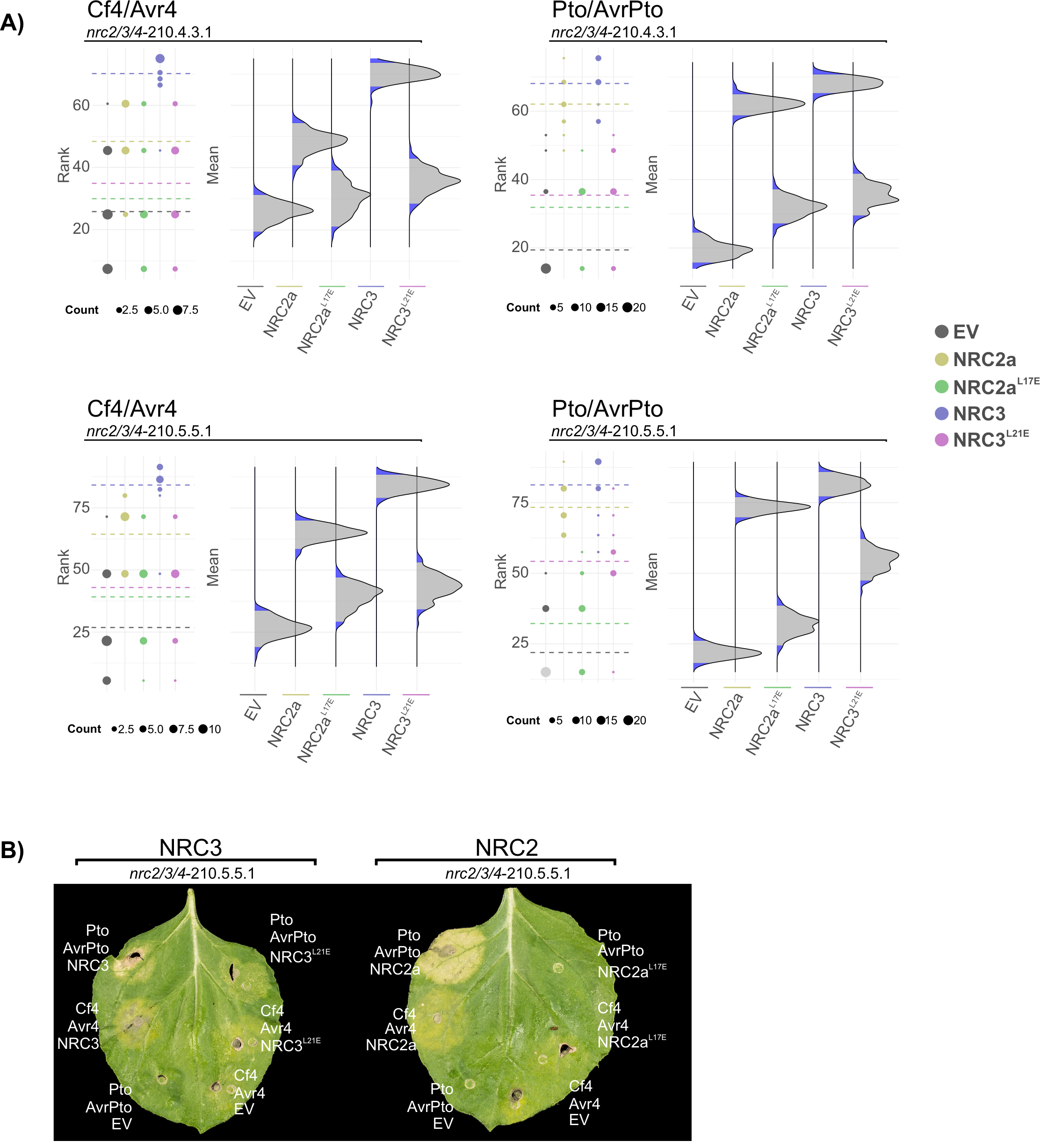

Supplement: S7 Fig — A) Statistical analysis was conducted using besthr R package [72]. The dots represent the ranked data and their corresponding means (dashed lines), with the size of each dot proportional to the number of observations for each specific value (count key below each panel). The panels on the right show the distribution of 100 bootstrap sample rank means, where the blue areas under the curve illustrate the 0.025 and 0.975 percentiles of the distribution. A difference is considered significant if the ranked mean for a given condition falls within or beyond the blue percentile of the mean distribution of the wild-type control. B) NRC MADA mutants cannot complement Cf-4/Avr4 or Pto/AvrPto-triggered hypersensitive cell death in the N. benthamiana nrc2/3/4 CRISPR lines. Representative N. benthamiana leaves infiltrated with appropriate constructs were photographed 7–10 days after infiltration. The NRCs tested, NRC2 and NRC3, are labelled above the leaf of NRC CRISPR line nrc2/3/4-210.5.5.1. The receptor/effector pair tested, Cf-4/Avr4 and Prf (Pto/AvrPto), are labelled on the leaf image. To ensure the NRC MADA mutants were not autoactive they were expressed with either Cf-4 or Pto and an EV control was taken along. (TIF) [file pgen.1010414.s007.tif]

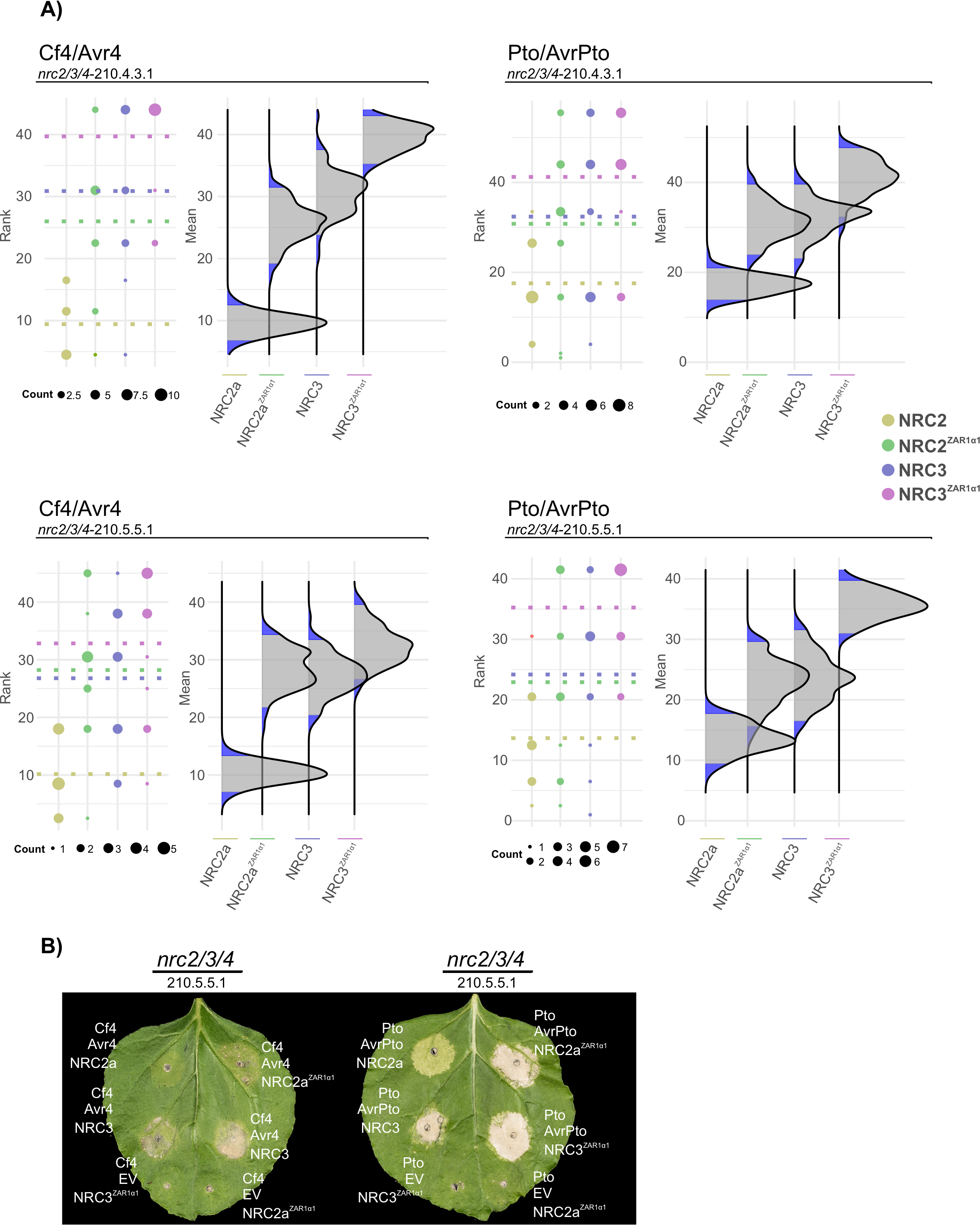

Supplement: S8 Fig — A) Statistical analysis was conducted using besthr R package [72]. The dots represent the ranked data and their corresponding means (dashed lines), with the size of each dot proportional to the number of observations for each specific value (count key below each panel). The panels on the right show the distribution of 100 bootstrap sample rank means, where the blue areas under the curve illustrate the 0.025 and 0.975 percentiles of the distribution. A difference is considered significant if the ranked mean for a given condition falls within or beyond the blue percentile of the mean distribution of the wild-type control. B) NRC ZAR1 α1 helix chimaeras can complement Cf-4/Avr4 and Pto/AvrPto-triggered hypersensitive cell death in the N. benthamiana nrc2/3/4 CRISPR lines. Representative N. benthamiana leaves infiltrated with appropriate constructs were photographed 7–10 days after infiltration. The receptor/effector pair tested, Cf-4/Avr4 and Prf (Pto/AvrPto), are labelled above the leaf of NRC CRISPR line nrc2/3/4-210.5.5.1. The NRCs tested, NRC2 and NRC3, are labelled on the leaf image. To ensure the NRC ZAR1 α1 helix chimaeras were not autoactive they were expressed with either Cf-4 or Pto and an EV control was taken along. (TIF) [file pgen.1010414.s008.tif]

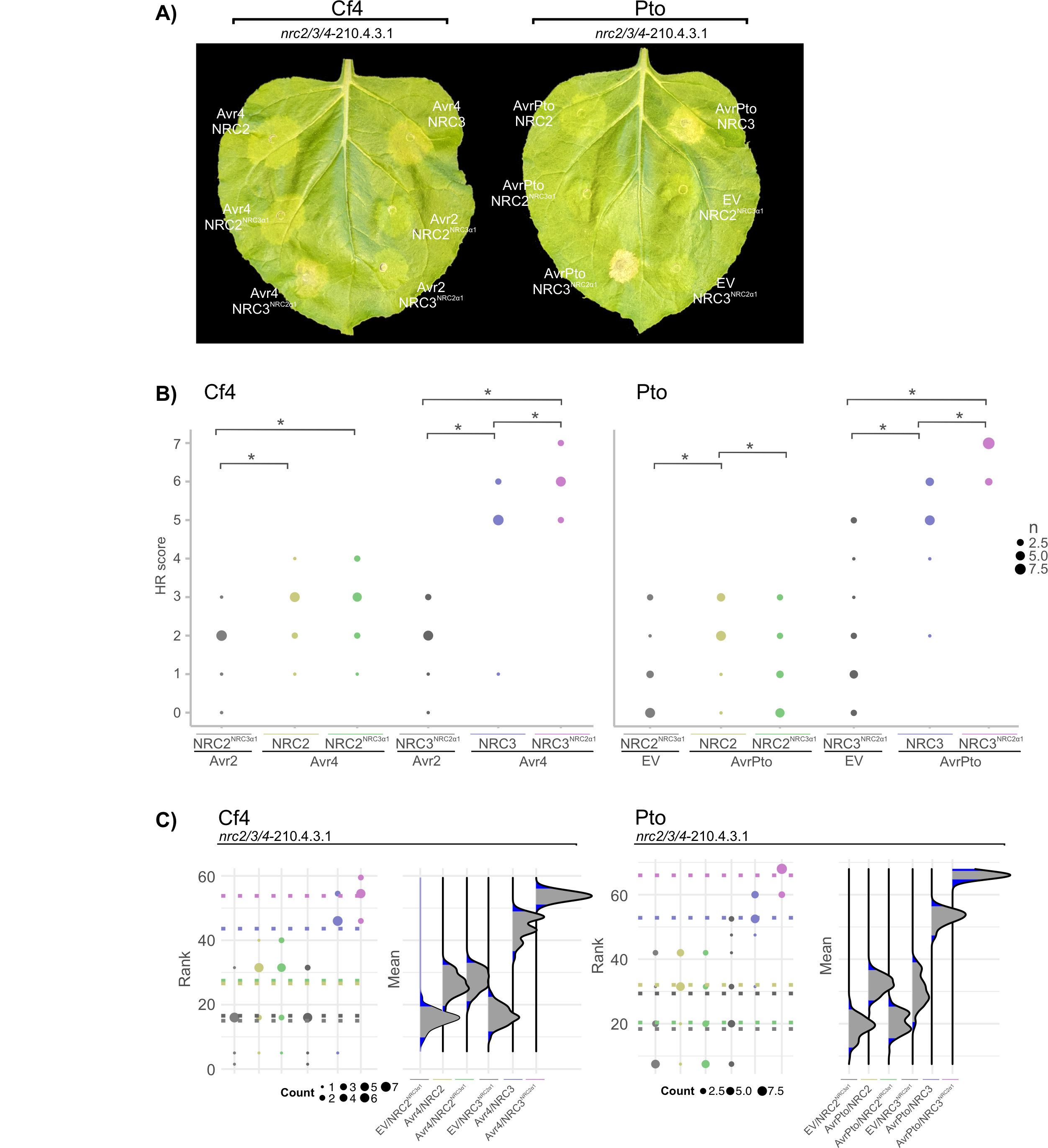

Supplement: S9 Fig — A) Residues 1–17 of NRC2 and 1–21 of NRC3 were replaced with residues 1–21 of NRC3 and 1–17 of NRC2 to generate the NRC2NRC3α1 and NRC3NRC2α1 chimaeras. NRC3 with the NRC2 α1 helix (NRC3NRC2α1) can complement Cf-4/Avr4 and Pto/AvrPto-triggered hypersensitive cell death in the N. benthamiana nrc2/3/4 CRISPR lines, while NRC2 with the NRC3 α1 helix (NRC2NRC3α1) cannot. Representative N. benthamiana leaves infiltrated with appropriate constructs were photographed 7–10 days after infiltration. The receptor tested, Cf-4 and Prf (Pto), is labelled above the leaf of NRC CRISPR line nrc2/3/4-210.4.3.1. The NRC and NRC chimaeras tested as well as the effectors are labelled on the leaf image. To ensure the NRC α1 helix chimaeras were not autoactive when expressed with either Cf-4 or Pto and an Avr2 or EV control was taken along, respectively. B) Quantification of hypersensitive cell death. Cell death was scored based on a 0–7 scale between 7–10 days post infiltration. The results are presented as a dot plot, where the size of each dot is proportional to the count of the number of samples. Significant differences between the conditions are indicated with an asterisk (*). C) Statistical analysis was conducted using besthr R package [72]. The dots represent the ranked data and their corresponding means (dashed lines), with the size of each dot proportional to the number of observations for each specific value (count key below each panel). The panels on the right show the distribution of 100 bootstrap sample rank means, where the blue areas under the curve illustrate the 0.025 and 0.975 percentiles of the distribution. A difference is considered significant if the ranked mean for a given condition falls within or beyond the blue percentile of the mean distribution of the wild-type control. (TIF) [file pgen.1010414.s009.tif]

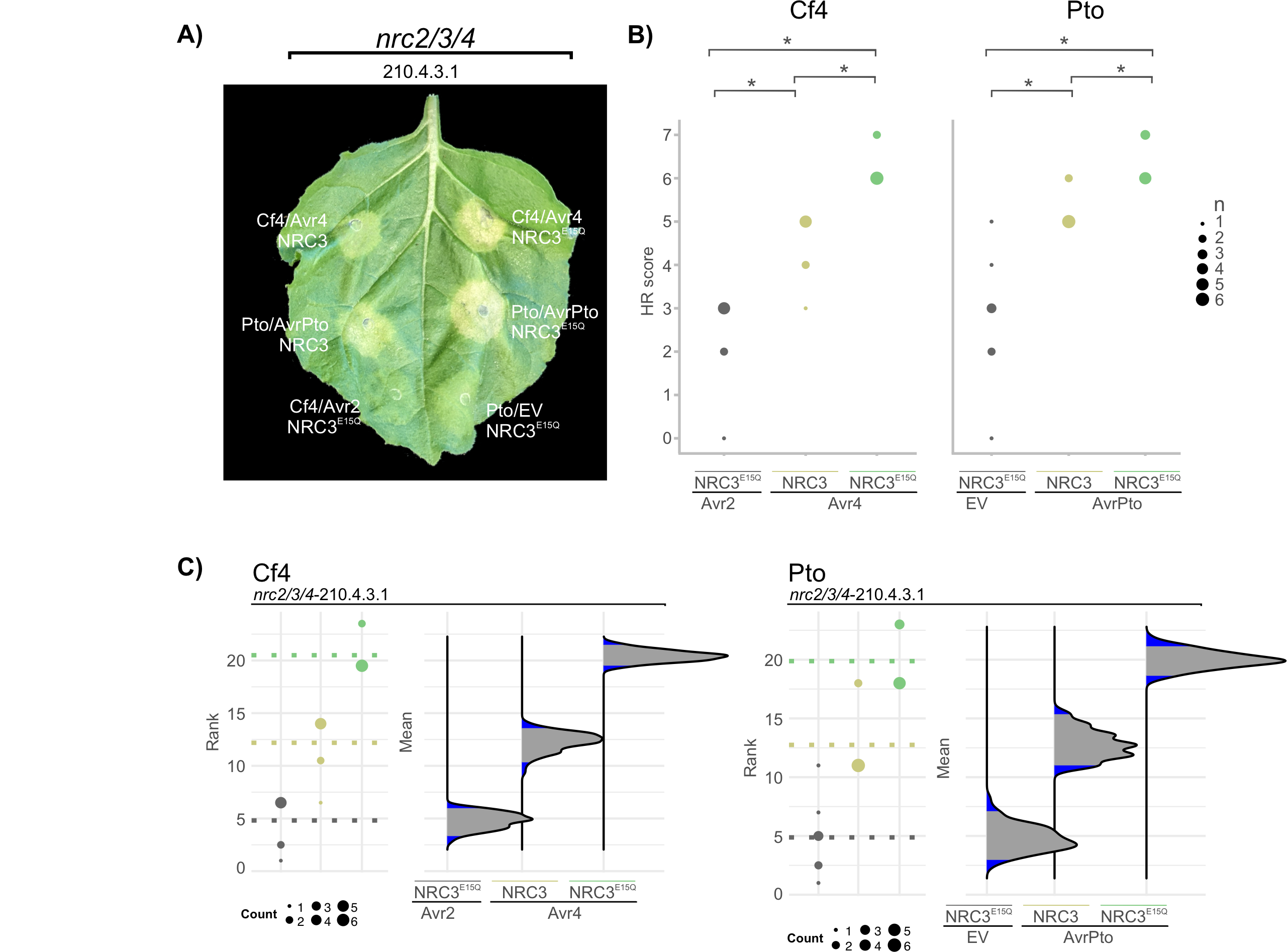

Supplement: S10 Fig — A) NRC3 and the NRC3 E15Q mutant can complement Cf-4/Avr4 and Pto/AvrPto-triggered hypersensitive cell death in the N. benthamiana nrc2/3/4 CRISPR lines. Representative N. benthamiana leaves infiltrated with appropriate constructs were photographed 7–10 days after infiltration. The receptor/effector pair tested, Cf-4/Avr4 and Prf (Pto/AvrPto), as well as the NRC and NRC mutants used are labelled on the leaf image. To ensure the NRC3 E15Q mutant was not autoactive we expressed it with either Cf-4 or Pto and an EV control. B) Quantification of hypersensitive cell death. Cell death was scored based on a 0–7 scale between 7–10 days post infiltration. The results are presented as a dot plot, where the size of each dot is proportional to the count of the number of samples with the same score. Significant differences between the conditions are indicated with an asterisk (*). C) Statistical analysis was conducted using besthr R package [72]. The dots represent the ranked data and their corresponding means (dashed lines), with the size of each dot proportional to the number of observations for each specific value (count key below each panel). The panels on the right show the distribution of 100 bootstrap sample rank means, where the blue areas under the curve illustrate the 0.025 and 0.975 percentiles of the distribution. A difference is considered significant if the ranked mean for a given condition falls within or beyond the blue percentile of the mean distribution of the wild-type control. (TIF) [file pgen.1010414.s010.tif]

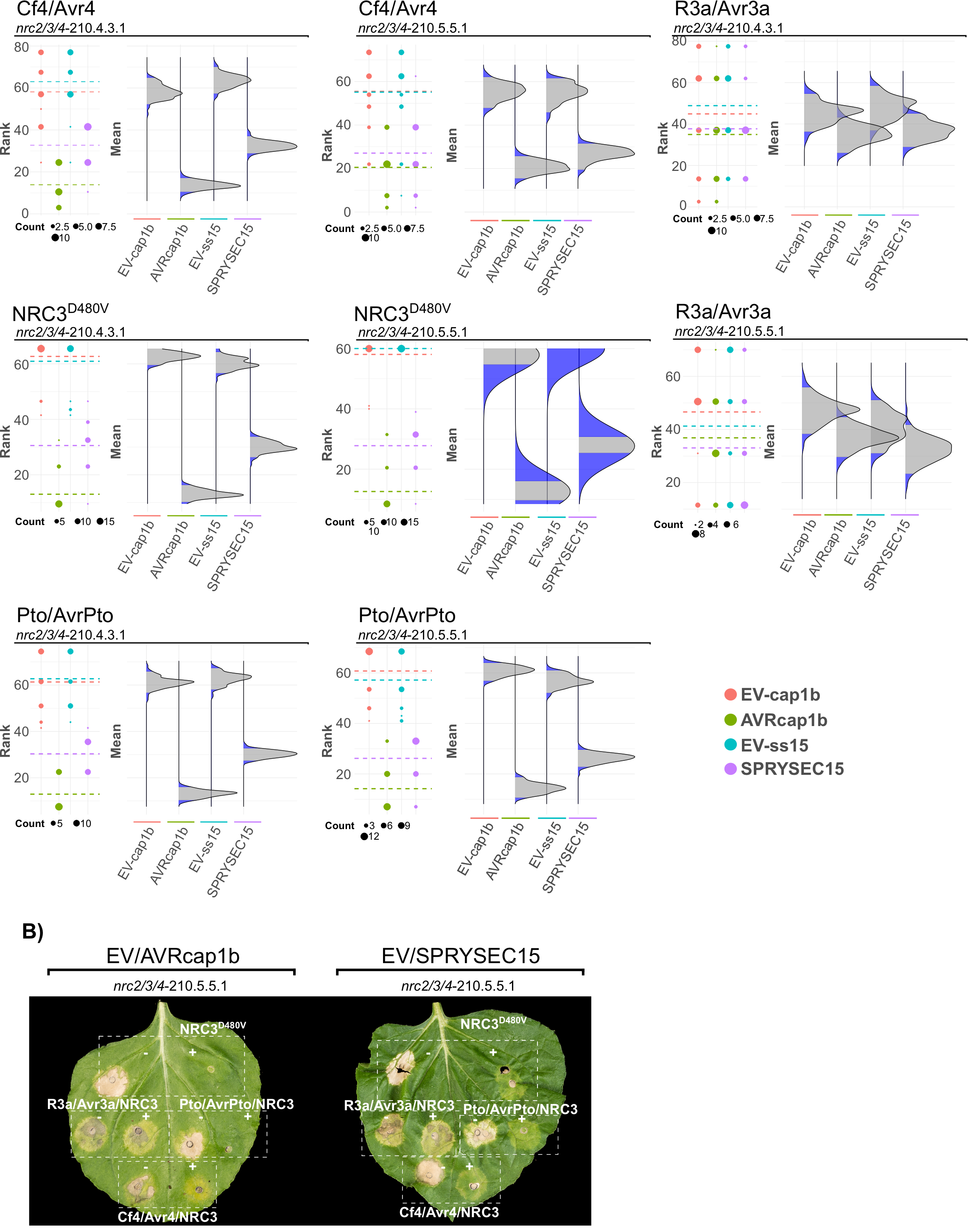

Supplement: S11 Fig — A) Statistical analysis was conducted using besthr R library [72]. The dots represent the ranked data and their corresponding means (dashed lines), with the size of each dot proportional to the number of observations for each specific value (count key below each panel). The panels on the right show the distribution of 100 bootstrap sample rank means, where the blue areas under the curve illustrates the 0.025 and 0.975 percentiles of the distribution. A difference is considered significant if the ranked mean for a given condition falls within or beyond the blue percentile of the mean distribution of the wild-type control. B) Cf-4/Avr4-triggered hypersensitive cell death is suppressed by SPRYSEC15 and AVRcap1b, but not the EV control. Representative image of N. benthamiana nrc2/3/4-210.5.5.1 CRISPR line leaves which were agroinfiltrated with NRC3/suppressor constructs, as indicated above the leaf, and either autoactive NRC3D480V, Prf (Pto/AvrPto), R3a/Avr3a, or Cf-4/Avr4 as labelled on the leaf image, photographed 7–10 days after infiltration. (TIF) [file pgen.1010414.s011.tif]
