# Supplementary material for: The helper NLR immune protein NRC3 mediates the hypersensitive cell death caused by the cell-surface receptor Cf-4
Source: PLoS Genet. 2022 Sep 22;18(9):e1010414. doi: 10.1371/journal.pgen.1010414 (PMC9543701; doi:10.1371/journal.pgen.1010414)
Supplement: Attachment — Submitted filename: 20220526_PGENETICS-D-22-00212_Rebuttal_V3.pdf [file pgen.1010414.s015.pdf]

Dear Dr. Qi and Köhler,

Thank you very much for your evaluation of our manuscript "The helper NLR immune protein NRC3 mediates the hypersensitive cell death caused by the cell-surface receptor Cf-4" for consideration as a research article at PLOS Genetics.

We appreciate the overall positive feedback of the the independent reviewers. We have endeavoured in this revision to address as much as possible the pertinent points they raised. Please see the point-by-point 'Response to Reviewers' attached to this file.

We hope that we have addressed many of the issues that were raised and we look forward to your response.

Best regards,

Sophien Kamoun
Group Leader and Professor

****

Response to Reviewers

Comments from the Associate Editor:

Generally, this work provides valuable data supporting NRC3 function in Cf4/Avr4-induced cell death, which will be of great interest for the field. However, I agree with several of the reviewers' comments that should be addressed to improve the manuscript. Specifically, I consider the following points necessary for improving the present manuscript: 1) As reviewer 3 suggests, analyzing variation pattern of helper NRCs, studying the α1 helix feature and MADA motif function will help to understand NRCs protein function. It would furthermore be important to determine NRC3's specificity in Cf4/Avr4-induced cell death. The data show that the ZAR1 α1 helix substitution for the NRC2 N-terminal equivalent sequence allows NRC2 to activate Cf4/Avr4-triggered cell death. It would be important to address whether the α1 helix of NRC3 determines its functional specificity. Furthermore, it would be relevant to test whether NRC3's α1 helix substitution can also allow NRC2 to activate Cf4/Avr4-triggered cell death.

We thank the editor and reviewers for these suggestions. We have now included the tomato and pepper NRC1/2/3 homologs, showing that also tomato and pepper NRC3, but not NRC1 or NRC2, can complement Cf-4/Avr4 HR in *N. benthamiana nrc2/3/4* CRISPR lines (**NEW** Figure S5). This indicates that NRC3 function downstream of LRR-RPs is functionally conserved in the Solanaceae. In addition, we have added complementation assays using reciprocal swaps of the NRC2 and NRC3 α1 helix (**NEW** Figure S9). We show that NRC3 with the NRC2 α1 helix can complement Cf-4/Avr4 HR in *N. benthamiana nrc2/3/4* CRISPR lines to an even greater extent than wild-type NRC3, while NRC2 with the NRC3 α1 helix cannot complement Cf-4/Avr4 HR. This indicates that the specificity is not encoded solely in the α1 helix. This is further indicated by the fact that the ZAR1 α1 helix can functionally substitute

the NRC3 α1 helix in NRC3 (Figure 4). How NRC2 with the ZAR1 α1 helix, but not NRC3 α1 helix can complement Cf-4/Avr4 HR in *N. benthamiana nrc2/3/4* CRISPR lines remains to be shown.

2) More evidences are needed, as reviewer 1 suggests, to show that NRCs may function similarly with ZAR1 as cation channel and form resistosomes. It would strengthen the manuscript if additional experimental evidence (e.g. testing whether point mutations of conserved amino acids within NRC3 affect its function, whether NRC3 oligomerizes after Cf4/Avr4 activation, whether NRC3 relocates to the plasma membrane after Cf4/Avr4 activation) would be provided to illustrate the molecular function of NRC3 on mediating Cf4/Avr4-induced cell death.

We have now added data showing that mutating NRC3 E15Q does not affect Cf4/Avr4 or Pto/AvrPto-dependent HR (**NEW**, figure S10). This is similar to what we had already shown for NRC4, where NRC4 E11A is still functional (Adachi *et al.*, 2019). While these acidic residues are required for ZAR1 Ca^{2+} function, these residues are not conserved in NRCs, and NRC2 for example has Q11. In addition, the recent Sr35 resistosome structure (Förderer *et al.*, 2022) highlights that it is likely that all CC-NLRs with an executor role function as Ca^{2+} channels. However, the acidic residues found in ZAR1 are *also* not required or conserved for the Ca^{2+} channel function of Sr35 (Förderer *et al.*, 2022). Instead, these residues are uniquely required for ZAR1 function.

NLR oligomerization assays are at their infancy and have only been established in a few labs and for a few NLR proteins. We have shown in recent preprints that NRC2 also oligomerizes into a likely resistosome upon activation by various NRC-sensors (Contreras *et al.*, 2022; Ahn *et al.*, 2022). We used NRC2 for these assays as it is amenable to biochemical assays due to high protein accumulation and its compatibility with the extraction methods. NRC3 unfortunately is so far not compatible with BN-PAGE. We have added a western showing NRC1/2/3/4 accumulation (**NEW**, figure 2B). NRC3 accumulation is much lower as compared to the other NRCs, explaining why biochemical assays with this protein are very difficult. We tried BN-PAGE assays using optimized constructs containing both Cf-4 and NRC3 on the same T-DNA, as well as switching to tomato or pepper NRC3. Under none of these conditions were we able to observe NRC3 resistosome formation. It also remains a possibility that there are other unknown players mediating the signal between Cf-4/Avr4, and NRC3, and without overexpression of these other players resistosome formation is unlikely to be observed using BN-PAGE. Nonetheless, given our results with NRC2 resistosome formation upon activation, and the recent finding that both Sr35 and ZAR1 form similar structures, we think it is highly likely that NRC3 also functions by resistosome formation upon activation by Cf-4/Avr4.

Instead, we focussed further on the genetics of signalling downstream of Cf-4/Avr4. We now show that EDS1 is dispensable for HR triggered by Cf-4/Avr4 (**NEW** Figure S2). Additionally, we now show that NRC3 can complement not only Cf-4/Avr4 HR in *N. benthamiana nrc2/3/4* CRISPR lines, but also Cf-2/Rcr3/Avr2, Cf-5/Avr5, and Cf-9/Avr9 HR (**NEW** Figure S6). Cf-4 and Cf-9 are homologs, but Cf-2 and Cf-5 belong to a distinct LRR-RP subclade (Kourelis *et al.*, 2020). This data combined with the fact that tomato and pepper NRC3 can complement Cf-4/Avr4 HR in *N. benthamiana nrc2/3/4* CRISPR lines highlights the important

hub function of NRC3 downstream of LRR-RP signalling in Solanaceae. In the text we now also more clearly indicate the fact that NRC3 is found in all Solanaceae genomes we looked at.

We anticipate that the additional mutants and genetics we have added to the manuscript should make this manuscript ready for publication. Please note also that we have carefully chosen to submit the paper to PLOS Genetics given that we emphasize the precise dissection of a complex genetic network using reverse genetics and complementation assays rather than biochemical experiments.

Reviewer's Responses to Questions

Comments to the Authors:
Please note here if the review is uploaded as an attachment.

Reviewer #1: This study is examining the involvement of NRC2/3 and 4 in cf-4 signaling. It was already known that NRCs are involved in Cf-4 cell death but the current study brings more detailed information on the specific contribution of NRC3 and to a lesser extent NRC2 to Cf-4 signaling. However, only cell death is evaluated, using a semi-quantitative scale, and in an overexpression system. This analysis is limited and could be complemented with a quantitative analysis of cell death and defense gene expression.

We thank the reviewer for the summary. Indeed, we focus on cell-death as one of the key NRC3-dependent outputs. The results we present are robust, repeated multiple independent times, and verified in independent manners, e.g. lack of cell death in CRISPR lines, complementation with NRC3, lack of complementation with functional mutants, and suppression of cell death by suppressors of NRC cell death. Any form of “more” quantitative cell death would still rely on overexpression of these proteins and hence is not an independent verification. Finally, as we highlight in the text there are also NRC3-independent responses which do not result in cell-death. As such, defence gene expression may or may not be affected in an NRC-dependent manner, but this is beyond the scope of the current manuscript.

The authors then show that NRC3 cell death is suppressed by mutations affecting some conserved residues in N-term and that ZAR1 N-term could rescue the phenotype, suggesting that NRCs and ZAR1 function similarly. This data does not show that NRC3 could be a cation channel. However, simple experiments could greatly enhance the novelty and quality of the study. ZAR1 E11, which is specifically involved in calcium permeation, is conserved in NRC3. Does mutating this residue to a Q in NRC3 affect Cf-4 cell death (or NRC3auto-activity)? Is NRC3 cell death inhibited by calcium channel blockers? Does auto active NRC3 trigger calcium influx and if yes, does it requires E14? If those experiment were conducted, they would link NRC3 to the regulation of calcium level which would be a considerable advance for the understanding of NRC3 function. Importantly, the text needs extensive editing to increase clarity and precision, in particular in the introduction and discussion.
If those changes were made, this study would be very valuable to the field.

We have now added data showing that mutating NRC3 E15Q does not affect Cf4/Avr4 or Pto/AvrPto-dependent HR (**NEW**, figure S10), see above response to the editor. As we explain, the acidic residues found in ZAR1 are uniquely required for ZAR1 function, and do not seem to be functionally conserved in other CC-NLRs. Therefore, this result does explicitly not exclude NRCs functioning as cation channels. In two independent manuscripts now on bioRxiv (Contreras *et al.*, 2022; Ahn *et al.*, 2022) we show that NRCs also oligomerize into likely resistosome structures upon activation. Unfortunately, due to the technical difficulties of working with NRC3 we have not been able to show this yet for NRC3. Similarly, the NRC-independent response likely also involves Ca^{2+} signalling, making it difficult to evaluate the role of NRC3 as a cation channel during cell-surface receptor mediated signalling.

Major comments:
Abbreviated terms must be spelled at first use. I noticed ZAR1, RPM1, ADR1, NRG1, MAPK etc

Spelled out NOD, ROS, MAPK, ADP, ATP, ZAR1, NRG1, ADR1, RPP1, Roq1, NADase, LYK5, CERK1, FLS2, SERK3/BAK1, SOBIR1, RRS1, RPS4, RPS2, RPS5, RPM1, EDS1, PAD4, and SAG101 on first occurrence.

The introduction should be made more accessible to non-specialist, for instance:
"In Arabidopsis, the EDS1/PAD4/ADR1 and, to a lesser extent, EDS1/SAG101/NRG1 modules are genetically required for a subset of the immune responses triggered by LRR-RPs" and latter "Since the EDS1/PAD4/ADR1 and EDS1/SAG101/NRG1 modules are typically associated with hypersensitive cell death signaling, this raises the question of what the molecular mechanism of this interaction is." These proteins have not been introduced and defined before. Why would we expect these modules to be involved in cf-4 cell death? Is it canonical for PRR to trigger cell death? A non-specialist reader cannot understand.
The introduction also should be more focused and clearly present the background knowledge required to understand the motivation and results of the study. I would appreciate a reminder of which NRC is required for which sensor NLR function. Extensive editing is required.

We rewrote the section on the requirement of EDS1/PAD4/ADR1 for cell-surface receptor signalling. We now also show that EDS1 is not required for Cf4/Avr4 cell-death in *N. benthamiana* (**NEW** Figure S2). For the purpose of this paper we think it is sufficient to keep the information on which sensor requires which NRC to a minimum as otherwise the introduction would become too long. Also, this would detract from the main focus of the paper.

Line 76 "specific variant cyclic ADP-ribose (v-cADPR)" The study of Yu D. et al 2021 (TIR domains of plant immune receptors are 2′,3′-cAMP/cGMP synthetases mediating cell death) should be mentioned.

We added this study. See line 85-88.

Line 169 "NRC3 is conserved in the Solanaceae and likely mediates hypersensitive cell death triggered by a variety of leucine-rich repeat receptor-like proteins. We propose that NRC3 is

a core node that connects cell surface and intracellular immune networks" What other receptors did you study here? What, in this study, justify such a broad proposition?

As highlighted in the response to the editor we now include pepper and tomato NRC3 (**NEW** Figure S5), and the LRR-RPs Cf-2, Cf-5, and Cf-9 (**NEW** Figure S6).

Figure 4: precise the residues used for the constructs in the figure, the caption and the corresponding results section. The author should be very cautious in the interpretation of the data as nothing tells us that the cell death triggered by the WT NRC3 and the chimeric NRC3 are the result of the same molecular mechanism. ZAR1 alpha1 helix is not sufficient to form a cation channel, the chimeric NRC3 data only indicates that NRC3 is still functional, even with ZAR1 alpha1 helix. Supporting data showing NRC3 regulates (directly or not) calcium levels in the cytoplasm and data showing that calcium influx is required for NRC3 function would be valuable. In addition, some residues in ZAR1 and in NRG1 and ADR1 have been shown to be specifically involved in calcium influx. Studying the orthologous residue in NRC3 would be a better indication that NRC3 forms a resistosome similar to ZAR1.

We have added this information in the text and figure caption. It seems likely now that most, if not all, CC-NLRs acting as helpers or singletons function as cation channels (Förderer *et al.*, 2022). We have also very recently shown that activated NRCs form higher-order oligomeric complexes (Contreras *et al.*, 2022; Ahn *et al.*, 2022). We therefore find it hard to imagine a scenario in which swapping the α1 helix of a CC-NLR can change the mode of signalling. The only thing that likely can be affected by mutations in the NLR is the activation signals or autoinhibitory states. As stated above, Ca^{2+} signalling likely also occurs independent of NRC3 downstream of cell-surface receptor mediated signalling, and therefore directly assessing this function of NRC3 is not experimentally feasible. Finally, we made E15Q mutations of NRC3 which do not affect its function, but as stated above that does not provide insights into Ca^{2+} signalling downstream of NRC3.

Minor comments
Introduction. Please avoid the use of unspecific terms like "generally" and "typically", be more precise. Also, avoid stating what "the community" thinks, please stick to the facts and let me make my own mind.

Where appropriate we have made the suggested edits.

I noticed (among others):
Line 38 "the parasite is blocked" precise

Changed blocked into: parasite invasion is restricted.

"The ligand sensing mechanisms are generally well-known" on line 52. This sentence does not bring information (generally) and is highly debatable (well-known).

Deleted.

Line 55 and 56 "both classes of receptors induce common signaling pathways" imprecise or incorrect

Replaced signalling pathways with outputs.

Line 60 "intracellular NLRs differs and converges is one of the main open questions in the field of plant immunity"

This has been defined as one of the main outstanding questions. As such we feel this sentence is appropriate to convey the importance to a more general audience.

Line 68 "Much progress has been made in recent years in understanding the molecular mechanisms of recognition and signaling of plant NLRs"

Changed much progress for several breakthroughs. This linking sentence highlights the importance to a more general audience. It is undeniable that the progress made in the last five years, which includes four structures of plant NLRs leading to important insights into their mode of activation and activity, important advances in elucidating TIR-produced metabolites and their roles in immunity, identifying CC-NLRs as cation channels, the first engineered plant NLRs, and the concept of NLR networks, constitute breakthroughs in the field of plant immunity.

Line 69 "In general,"

Now changed to NLR activation upon ligand recognition can result. Exceptions are a possibility. Indeed, RPP1 is ADP bound in the active form, although an ADP to ATP switch upon activation followed by subsequent hydrolysis is likely. Analysis of residues interacting with ADP/ATP indicate that most characterized NLRs are likely ATP bound in the active state (Ma *et al.*, 2020).

Line 144 "NRCs form redundant nodes in complex receptor networks, with different NRCs exhibiting different specificities for their sensor NLRs." So, NRCs are redundant and specific?

That is indeed the concept of an NLR network.

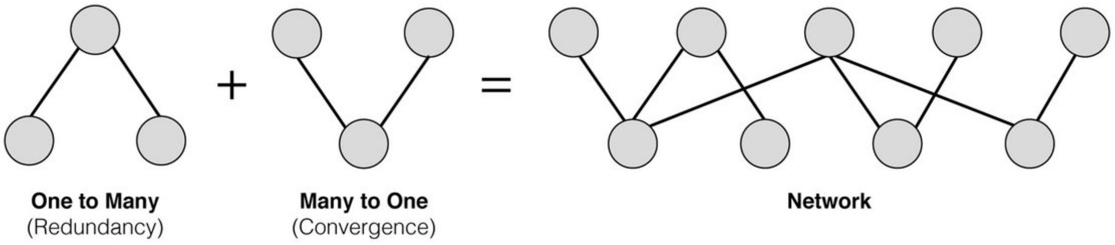

Line 160 "Using a combination of reverse genetics and complementation, we determined that Cf-4/Avr4-triggered cell death is largely mediated by NRC3, and not NRC1." NRC1? Which organism?

For further clarity we changed this to: Cf-4/Avr4-triggered cell death is largely mediated by the *N. benthamiana* NRC3 homolog, and not a *N. benthamiana* NRC1 homolog as previously implied.

A fragment of tomato NRC1 was used for VIGS in *N. benthamiana*. It is now clear that *N. benthamiana* does not have a NRC1 homolog. In addition, we also show that tomato (and pepper) NRC3, but not NRC1 or NRC2 can complement Cf-4/Avr4 in *N. benthamiana nrc2/3/4* lines.

Line 189 “indicating that the reduction in cell death is not correlated with Cf-4” imprecise.

Changed to: indicating that the reduction in cell death is not due to differences in Cf-4 protein accumulation.

Line 190 “Cf-4/Avr4 expression resulted in a specific chlorosis in the nrc2/3 and nrc2/3/4 lines which did not develop into confluent cell death (Figure 1C, Figure S1B).” What is specific about this chlorosis? Please go straight to the point.

Removed “a specific”.

Line 244 “only weakly complemented Cf-4/Avr4-triggered cell death” Avoid the use of unspecific terms.

Changed into: but only partially complemented Cf-4/Avr4-triggered cell death to as compared to NRC3.

Also changed other instances including the word weak.

Line 300 “NRC2L17E and NRC3L21E correspond to the NRC4L17E mutant” Imprecise

Changed into: L17E and L21E mutations in NRC2 ($NRC2^{L17E}$) and NRC3 ($NRC3^{L21E}$) correspond to the L17E mutation in NRC4 ($NRC4^{L17E}$).

Line 352 “In the activated ZAR1 resistosome, the N-terminal α1 helix of the CC domain forms a Ca2+-permeable membrane-permeable pore” delete the extra permeable.

Deleted the extra permeable.

Line 511 “Additionally, the ZAR1 α1 helix can functionally substitute for the N-terminal equivalent sequence of NRC3” This is said two sentence earlier

Deleted: and the ZAR1 α1 helix can substitute for the NRC3 α1 helix.

Line 521 “It may be that this chimeric protein is more “trigger happy”, either due to reduced autoinhibition or enhanced capacity to generate the resistosome funnel.” We can only say the chimeric construct is a gain of function. We don’t know if the effect is coming from increased activation (trigger happy) or more efficient activation of downstream signaling component.

At this point of the discussion some speculation is warranted. The words “It may be” clearly indicate speculation.

Reviewer #2: The author's group performed a series of work on the identification and characterization of helper NLRs in the NLR network. They discovered several helper NLRs including NRC2, NRC3 and NRC4 in Solanaceae plants that are required for the sensor NLR-mediated immune signaling (Wu et al., 2017, PNAS); They further screened a large amounts of effectors from diverse pathogens that can target and suppress the activity of NRCs, and revealed plant pathogens evolves to counteract the NRCs hub to subvert the NLR-mediated defense (Derevnina et al., 2021, PLoS Biology). This work further extends these studies by investigating the functional role of NRCs in pattern-triggered immunity (PTI) besides the intracellular effector-trigged immunity (ETI) as they reported previously. They establish a functional link between the cell death caused by PTI and the NLR resistosome. Overall, this work is interesting and further deepen our understanding of intricate immune receptor network in plants.

We thank the reviewer for the summary.

Major concerns

1. What is the biological significance of the convergent NRC hub? On one hand, it is proposed that it would be beneficial for the plant to defend against diverse pathogens by using one system. However, just like the double-edge, studies from the author's group indicated that the convergent NRC hub could be easily be targeted by the pathogens, leading to the disruption of the immune barrier. This is an arm race between plant and pathogens. Figure 5B， the author used some suppressors (AVRcap1b and SPRYSEC15) to further verify the specific role of NRC3 in Cf4/Avr4-induced cell death, the design of this experiments is good. I wonder whether these events could occur under natural conditions? Have the author observed some mixed infections of some pathogens in plants under natural conditions, and the plants displayed an increased symptom severity compared with infection of a pathogen alone? and does it associate with the suppression of the NRC hub?

Pathogen co-infection resulting in overcoming non-host resistance is well documented for coinfection of *Albugo* and *Phytophthora infestans* in Arabidopsis or with viral pathogens. Additionally, many effectors are shown to target hub proteins such as SERK3/BAK1, thereby inhibiting host immune responses. It is therefore possible that co-infections can lead to gain of virulence of some pathogens. We have not investigated whether this occurs in Solanaceous plants by suppression of the NRC network.

2. How does or what is mechanism underlying these effectors-mediated suppression of NRC activity. In fact, there remains largely unknown whether AVRcap1b and SPRYSEC15 inhibit the the formation of resistosome or the subsequent immune signal transduction, the author may discuss this.

We now added the following sentence to the discussion: In the case of SPRYSEC15 this suppression involves direct binding of the effector to the NB-ARC domain of NRC proteins, while the suppression mediated by AVRcap1b is indirect.

The exact mechanism of suppression by these effectors is the subject of ongoing studies in the lab.

3. What is the relationship between Cf-4 and NRC3? Does Cf-4 interacts with NRC3?

We have not established whether the NRC3 and Cf4 biochemically interact, or how the signal downstream of Cf4 is relayed to NRC3. Cf-4 also requires SOBIR1 to trigger HR. It is likely that there are other proteins required as well. In line 434-436 we speculate on this, and this is indicated by the question marks in our final model (Figure 6).

Reviewer #3: Kourelis and colleagues present the work investigating the role of helper NLR, NRCs in N. benthamiana, in mediating HR triggered by the cell-surface receptor Cf-4. Genetic components required for Cf-4/Avr4-mediated HR had been analyzed in N. benthamiana using VIGS (Gabriëls, Takken et al., 2006, MPMI), identifying an NLR, which was named as NRC1 back then. Given that there are recent advances in the field on uncovering the role of helper NLRs (such as ADR1-EDS1-PAD4 by Pruitt et al.) serving as a converging point interconnecting signaling from surface-bound receptors and intracellular NLR receptors, the presented work on Cf-4 requiring one of the helper NLRs previously characterized in Solanaceae is quite timely to corroborate the helper's role in immune signaling. This work utilized the previously generated stable transgenic lines of N. benthamiana carrying mutations in multiple NRC genes and wisely employed the transient complementation system to reintroduce individual NRC genes as wild-type or mutated versions to address the functional specificity. The authors successfully identified NRC3 as the main contributor of the Cf-4-mediated HR, while its homolog NRC2, despite no complementation as a wild-type construct, could be expanded to contribute to the Cf-4-mediated HR signaling when the alpha1 helix was replaced with the one from Zar1. The authors also employed known effectors that suppress the helper NRCs to negate the function of NRC3 participating to Cf-4-mediated HR. These findings collectively suggest that the helper NLR function and its specificity in the participating signaling pathways can be modified for future genetic engineering. All these conclusions were made with quantitation of the transient HR assays in N. benthamiana along with the confirmation of protein expression at adequate level.
This work is timely addition to the field in characterizing the signaling potential of helper NLRs.

We thank the reviewer for the summary of the paper.

Generally, I do not find major flaw or amendment points, however, I wish I could find robust discussion on potential mechanistic explanation on this convergence. Especially, NRC3, which is claimed to be conserved, shall be discussed in terms of the variation pattern as compared with other helper NRCs. As the alpha 1 helix feature appears to matter, and MADA motif is rather loose and remains to be further defined, this work may have a saying in refining the functional MADA motif. In this sense, I would propose to look into sequence alignments of NRCs (or provide previous works related to this) in various species to further define functional motifs in NRCs, such as pinpointing the variation pattern in the MADA motif.

In line 194, 263, 455, 465 and figure S3 we highlight that *NRC3* is conserved in the Solanaceae. In addition, we now show that tomato and pepper NRC3, but not tomato and

pepper NRC1 or NRC2 can complement Cf-4/Avr4 cell death in *N. benthamiana nrc2/3/4* lines (**NEW** Figure S5). NRC2 and NRC4, like NRC3, are also conserved in the Solanaceae. Therefore, there is no clear distinction in conservation of NRC3 as compared to other NRCs. The function of NRC3 to partner with cell surface receptors, however, does seem to be conserved.

We now show that NRC3 is still functional with both the ZAR1 and NRC2 α1 helix. Therefore, the specificity for signalling downstream of Cf-4/Avr4 is not determined by the α1 helix alone. How *N. benthamiana* NRC2 with the ZAR1 α1 helix, but not NRC3 α1 helix, has an expanded function as helper-NLR is not clear. Several of the α1 helix mutations we tested in this manuscript (α1 helix swaps and E15Q mutation) seemingly enhanced the cell-death response. While *N. benthamiana* NRC2 can complement Cf4/Avr4, albeit to a much lesser extent than NRC3, tomato and pepper NRC2 could not. The enhanced response of some of the α1 helix mutations could explain why we were then able to see clear complementation. It is however highly likely that regions other than the α1 helix are involved in determining helper-NLR specificity.

In addition, I propose to include exact positions of alpha1-swapping with numbered AA positions in Figure 4 A and B. I do not find that information in the main text or in main method section. For example, the statement in L355-356 and Figure 4A do not match; As Figure 4A does not show the sequence alignment, but structure, it would be impossible to see the homology in the presented figure.

We now include details of the position of the swap in the main text and figure legend.

In the following section, I provide comments that the authors could use to tighten the loose ends in their interpretation of datasets or fix typographic errors.

L140: R genes encoding (instead of encoded) sensor NLRs

Changed into: consists of sensor NLRs encoded by *R* genes which

L149: add space after [42]

Space added.

L157: required for downstream signaling upon LRR-RP activation

Changed as suggested.

L162: As NRC1 from tomato was introduced in L133 as a Cf-4 signaling factor, it would be relevant to specify the NRCs (NRC3 and NRC1) are from N. benthamiana.

Changed into: Cf-4/Avr4-triggered cell death is largely mediated by the *N. benthamiana* NRC3 homolog, and not a *N. benthamiana* NRC1 homolog as previously implied

L178: It would be relevant to state clearly here that the lines employed in this study were generated previously. The current sentence reads as if these were generated for this work despite the references.

Added the word previously.

Listing specific lines available (or used) would also help, instead of stating it as "various" combinations. Only three different lines were used in this work, which should be relevant to introduce one by one.

We added this in the next sentence.

L239: italicizes Sl in SlNRC1 if the authors want to be consistent with L245.

Italicized.

Figure 3A: The alignment of NRCs with ZAR1 seems not well spaced, for example, I in NRC3 is not positioned in the middle of the space and the VV residues in Zar1 is squeezed so that the second V is cut off at the end. Using the font courier may solve this problem.

We thank the reviewer for spotting this mistake, which occurred by automatic font switching upon adding the alignment into CorelDRAW. We have now corrected this, maintaining the monospaced font.

L291-311: experiments were carried out both with NRC2 and NRC3 to test the function of MADA but the conclusion was only made for NRC3 in L310-311. Please clarify the use of NRC2L17E constructs in line of NRC3 results.

Changed the conclusion into: We conclude that the N-terminal α1 helix of NRC2 and NRC3 is required for their function as helper-NLRs.

I also wondered where the previous notion (L307) is to be found. It would be necessary to add a pointer to indicate actual dataset presented in a previous figure (Figure 2 and 3S?).

Added the reference to the figures.

L351-352: "permeable" is duplicated. Would it be the best phrasing for describing the Zar1 action?

We have removed this duplication. As the exact mechanism remains to be fully elucidated permeable is the most agnostic word as to the exact mechanism.

L369-370: This conclusion should be more specific than the current simple version. The chimeric constructs do not merely "replace" but apparently augmented the function in NRC2 in particular. The authors have a tendency of making conclusions of NRC2 and NRC3 together, while there are distinct spectrum in helper function. This tendency of generalizing should be fixed.

Changed the conclusion of this section into:

We conclude that the α1 helix of ZAR1, which is known to make a Ca^{2+}-permeable membrane pore, can replace the NRC2 and NRC3 α1 helix for hypersensitive cell death signaling, and even enhance the NRC-dependent hypersensitive cell-death response. Why the $NRC2^{ZAR1\alpha1}$ chimaera, but not the $NRC2^{NRC3\alpha1}$ chimaera, can complement Cf-4/Avr4-triggered cell death to a similar extent as NRC3 is not clear. We conclude that modifications of the α1 helix of NRCs can sometimes expand their role as helper-NLRs.

L419: Be consistent with citation format (numbers in bracket is commonly found).

Properly inserted these references.

L494: The same comment is given to the authors as L162. Please specify species name for the previous work on NRC1. If the previous naming is confusing (indeed it is referring to an enigmatic entity due to VIGS), the nomenclature is to be corrected where relevant and the usage of NRC1 should be avoided thereafter.

Slightly changed the sentence into: Previously, NRC1 was implicated in the cell death mediated by the cell surface receptors Cf-4, LeEIX2 and Ve1 in *N. benthamiana*. Here we show that NRC3, rather than NRC1, links cell-surface immune receptors to intracellular immunity in the *N. benthamiana* experimental system (**Figure 1, 2**).

We now show multiple NRC3 homologs can complement Cf4/Avr4 in *N. benthamiana*, but not NRC1 homologs from either tomato or pepper (**NEW** Figure S5). That *N. benthamiana* in fact does not contain a *NRC1* homolog does not matter to the conclusion, which is that no NRC1 homologs can complement Cf4/Avr4 in *N. benthamiana nrc2/3/4*.

Have all data underlying the figures and results presented in the manuscript been provided?

Large-scale datasets should be made available via a public repository as described in the *PLOS Genetics* data availability policy, and numerical data that underlies graphs or summary statistics should be provided in spreadsheet form as supporting information.

Reviewer #1: Yes

Reviewer #2: Yes

Reviewer #3: Yes

PLOS authors have the option to publish the peer review history of their article (what does this mean?). If published, this will include your full peer review and any attached files.

If you choose “no”, your identity will remain anonymous but your review may still be made public.

Do you want your identity to be public for this peer review? For information about this choice, including consent withdrawal, please see our Privacy Policy.

Reviewer #1: No

Reviewer #2: No

Reviewer #3: **Yes:** Eunyoung Chae

In compliance with data protection regulations, you may request that we remove your personal registration details at any time. (Remove my information/details). Please contact the publication office if you have any questions.
