# Supplementary material for: The helper NLR immune protein NRC3 mediates the hypersensitive cell death caused by the cell-surface receptor Cf-4
Source: PLoS Genet. 2022 Sep 22;18(9):e1010414. doi: 10.1371/journal.pgen.1010414 (PMC9543701; doi:10.1371/journal.pgen.1010414)
Supplement: Attachment — Submitted filename: PGENETICS-D-22-00212R1_Response_v2.pdf [file pgen.1010414.s016.pdf]

Dear Dr. Qi and Köhler,

Thank you very much for your evaluation of our manuscript "The helper NLR immune protein NRC3 mediates the hypersensitive cell death caused by the cell-surface receptor Cf-4" for consideration as a research article at PLOS Genetics.

We appreciate the overall positive feedback of the independent reviewers and have made the suggested textual changes. Please see the point-by-point 'Response to Reviewers' attached to this file.

We hope that we have addressed many of the issues that were raised and we look forward to your response.

Best regards,

Sophien Kamoun
Group Leader and Professor

From: PLOS Genetics <em@editorialmanager.com>
Date: 27 July 2022 at 19:16:17 GMT+2
To: "Sophien Kamoun (TSL)" <Sophien.Kamoun@tsl.ac.uk>
Subject: Decision on your article submitted to PLOS Genetics (PGENETICS-D-22-00212R1) - [EMID:2716a4e8b914cd01]
Reply-To: PLOS Genetics <plosgenetics@plos.org>

CC: "Jiorgos Kourelis" jiorgos.kourelis@tsl.ac.uk, "Mauricio P. Contreras" mauricio.contreras@tsl.ac.uk, "Adeline Harant" adeline.harant@tsl.ac.uk;adeline.harant@gmail.com, "Hsuan Pai" hsuan.pai@tsl.ac.uk, "Daniel Lüdke" daniel.luedke@tsl.ac.uk, "Hiroaki Adachi" adachi.hiroaki@bs.naist.jp, "Lida Derevnina" ld645@cam.ac.uk, "Chih-Hang Wu" wuchh@gate.sinica.edu.tw

Dear Dr Kamoun,

Thank you very much for submitting your Research Article entitled 'The helper NLR immune protein NRC3 mediates the hypersensitive cell death caused by the cell-surface receptor Cf-4' to PLOS Genetics.

The manuscript was fully evaluated at the editorial level and by independent peer reviewers. The reviewers appreciated the attention to an important topic but identified some concerns that we ask you address in a revised manuscript. Specifically, we agree with reviewer 1 that the present data are insufficient to suggest that NRC3 forms a resistosome and functions as a calcium channel like ZAR1. In a revised manuscript we therefore advice to tone down the related statements unless more experimental data is provided. The related descriptions like “resistosome” and “Ca2+ channel” concerning NRC3 molecular function should be weakened and revised including the model (Fig 6).

We therefore ask you to modify the manuscript according to the review recommendations. Your revisions should address the specific points made by each reviewer.

We thank the editors and reviewers for their contributions. In the revised manuscript we have toned down statements suggesting that NRC3 forms a resistosome and functions as a calcium channel like ZAR1 or Sr35. See below in response to reviewer 1 in more detail.

In addition we ask that you:
1) Provide a detailed list of your responses to the review comments and a description of the changes you have made in the manuscript.
2) Upload a Striking Image with a corresponding caption to accompany your manuscript if one is available (either a new image or an existing one from within your manuscript). If this image is judged to be suitable, it may be featured on our website. Images should ideally be high resolution, eye-catching, single panel square images. For examples, please browse our archive. If your image is from someone other than yourself, please ensure that the artist has read and agreed to the terms and conditions of the Creative Commons Attribution License. Note: we cannot publish copyrighted images.

We hope to receive your revised manuscript within the next 30 days. If you anticipate any delay in its return, we would ask you to let us know the expected resubmission date by email to plosgenetics@plos.org.

If present, accompanying reviewer attachments should be included with this email; please notify the journal office if any appear to be missing. They will also be available for download from the link below. You can use this link to log into the system when you are ready to submit a revised version, having first consulted our Submission Checklist.

While revising your submission, please upload your figure files to the Preflight Analysis and Conversion Engine (PACE) digital diagnostic tool. PACE helps ensure that figures meet PLOS requirements. To use PACE, you must first register as a user. Then, login and navigate to the UPLOAD tab, where you will find detailed instructions on how to use the tool. If you encounter any issues or have any questions when using PACE, please email us at figures@plos.org.

Please be aware that our data availability policy requires that all numerical data underlying graphs or summary statistics are included with the submission, and you will need to provide this upon resubmission if not already present. In addition, we do not permit the inclusion of phrases such as "data not shown" or "unpublished results" in manuscripts. All points should be backed up by data provided with the submission.

To enhance the reproducibility of your results, we recommend that you deposit your laboratory protocols in protocols.io, where a protocol can be assigned its own identifier (DOI) such that it can be cited independently in the future. Additionally, PLOS ONE offers an option to publish peer-reviewed clinical study protocols. Read more information on sharing protocols at https://plos.org/protocols?utm_medium=editorial-email&utm_source=authorletters&utm_campaign=protocols

Please review your reference list to ensure that it is complete and correct. If you have cited papers that have been retracted, please include the rationale for doing so in the manuscript text, or remove these references and replace them with relevant current references. Any

changes to the reference list should be mentioned in the rebuttal letter that accompanies your revised manuscript. If you need to cite a retracted article, indicate the article's retracted status in the References list and also include a citation and full reference for the retraction notice.

PLOS has incorporated Similarity Check, powered by iThenticate, into its journal-wide submission system in order to screen submitted content for originality before publication. Each PLOS journal undertakes screening on a proportion of submitted articles. You will be contacted if needed following the screening process.

To resubmit, you will need to go to the link below and 'Revise Submission' in the 'Submissions Needing Revision' folder.

Please let us know if you have any questions while making these revisions.

Yours sincerely,

Tiancong Qi
Academic Editor
PLOS Genetics

Claudia Köhler
Section Editor
PLOS Genetics

The revised manuscript has largely improved and addresses most issues raised by reviewers. However, I agree with reviewer 1 that the present data are insufficient to suggest that NRC3 forms a resistosome and functions as a calcium channel like ZAR1. In a revised manuscript the authors should therefore tone down the related statements unless more experimental data is provided. The related descriptions like "resistosome" and "Ca2+ channel" concerning NRC3 molecular function should be weakened and revised including the model (Fig 6).

Reviewer's Responses to Questions

Comments to the Authors:
Please note here if the review is uploaded as an attachment.

Reviewer #1: This study characterizes the involvement of NRCs in PRR-triggered cell death. As is, it is an interesting study presenting overall valuable data. Addition of the pepper and tomato NRC data is interesting and does extend the genetic characterization of the NRC family. The authors chose to only consider cell death as a proxi for NRC function and do not wish to investigate gene expression regulation. It would be interesting but I think it is fine since the conclusion related to NRC function are explicitly limited to cell death.

I think this manuscript could be published without additional experiments if the authors were not speculating on the molecular function of NRC3, resistosome formation and calcium influx, from the data presented in figure 4. The experimental approach used in figure 4 is limited and the data is overinterpreted.
I understand biochemical analyses of NLRs are technically challenging but if they are not conducted, the term resistosome should not be used to describe active NRC3 complexes. NLRs act through various mechanisms: holoenzyme formation, ion channel formation or by recruiting downstream signaling components. It is early to state that CNLs all act by forming ZAR1-like resistosomes from just two examples. The chimera experiment does not give us information on the WT protein function in the absence of data on oligomerization, membrane localization and calcium influx (ZAR1 alpha 1 helix is also required for plasma membrane localization so the ZAR1 helix could complement loss of proper subcellular localization of NRC3 mada mutant). Contrary to what is stated in the text (line 336), the alpha 1 helix does not form a pore, not alone. It only contributes to 1/3 of the ZAR1 channel. So, the chimeric constructs do not prove that NRC3 oligomerize in a resistosome-like oligomer. The fact that E14Q NRC3 mutant is not a loss of function (in these assays using overexpression and only considering cell death) does not suggest that NRC3 functions like ZAR1. Several other negatively charged residues are present in the alpha1 helix and could still be required for function. These could be tested alone or in combination. If NRC3 activity was correlated to calcium influx and if NRC3 ZAR1alpha1 E11Q mutant lost cell death, it would indicate that the function complemented by the alpha 1 helix is calcium influx. Then, I would cautiously admit that NRC3 likely forms a resistosome such as ZAR1. These experiments are not technically challenging.

We thank the reviewer for their comments and suggestions. We have toned down statements related to resistosome formation and Ca^{2+} channel activity as follows:

Introduction, line 193: “Using defined mutations in the MADA motif and by generating swaps with the Arabidopsis ZAR1 α1 helix, we show that NRC resistosome function is likely downstream of Cf-4 activation by Avr4.” -> “By using defined mutations in the MADA motif and generating swaps with the Arabidopsis ZAR1 α1 helix, we show that NRC resistosome function is potentially downstream of Cf-4 activation by Avr4.”

Introduction, line 191: “We conclude that Cf-4/Avr4-triggered hypersensitive cell death is mediated by the MADA-CC-NLR NRC3, likely requiring the formation of a Ca^{2+}-permeable pore.” -> “We conclude that Cf-4/Avr4-triggered hypersensitive cell death is mediated by the MADA-CC-NLR NRC3.”

Results section 4, line 337: “In the activated ZAR1 resistosome, the N-terminal α1 helix of the CC domain forms a Ca^{2+}-permeable membrane-pore -> “In the activated ZAR1 resistosome, the N-terminal α1 helix of the CC domain is an important determinant for cell death induction”

Results section 4, line 338: “In order to determine whether a similar mechanism also applies for Cf-4/Avr4-triggered hypersensitive cell death” -> “In order to determine whether the N-terminal α1 helix of the ZAR1 CC domain could substitute for the NRC2/NRC3 predicted α1 helix in Cf- 4/Avr4-triggered hypersensitive cell death”

Results section 4, line 362: “We conclude that the α1 helix of ZAR1, which is known to make a Ca^{2+}-permeable membrane pore” -> “We conclude that the α1 helix of ZAR1”

Discussion, figure 6, line 444: “This cell death requires an intact α1 helix of NRC3 indicating the formation of an activated NRC3 resistosome” -> “This cell death requires an intact α1 helix of NRC3 indicating the possible formation of an activated NRC3 resistosome”

Discussion, figure 6: arrow from NRC3 to resistosome and from resistosome to membrane as dashed arrows with question marks.

Discussion, line 478: “This observation suggests that the hypersensitive cell death response downstream of Cf-4/Avr4 immune signaling probably involves an activated resistosome.” -> “This observation suggests that the hypersensitive cell death response downstream of Cf-4/Avr4 immune signaling could involve an activated resistosome.”

Discussion, line 492: “Alternatively, the α1 helix may result in a more stable activated resistosome or form a more effective Ca^{2+} channel than the NRC3 N-terminus (see below).” -> “Alternatively, the ZAR1 α1 helix may stabilize activated NRC2.”

Discussion, line 499: “The recent observation that the ZAR1 α1 helix forms a Ca^{2+}-permeable membrane-permeable pore [7], and that the ZAR1 α1 helix can functionally substitute for the NRC2/3 N terminus, indicates that a Ca^{2+}-permeable membrane-permeable pore is possibly involved in mediating Cf-4/Avr4-triggered cell death.” -> “The recent observation that the ZAR1 α1 helix forms a resistosome that acts as a Ca2+-permeable membrane-permeable pore [7] raises the possibility that a similar mechanism mediates Cf-4/Avr4-triggered cell death.”

I noted 2 typos:
Line 259 : Cf-4/Avr4 cell death (Fig 2C and 2D, S4A Fig) for statistical analysis

We have corrected the brackets.

Line 345: Like wild type NRC3,

Changed all instances of “wild type” -> “wild-type”

Reviewer #2: The author has addressed my concern.

We thank the reviewer for their efforts to improve the paper.

Reviewer #3: The authors had successfully addressed major issues raised in the first round of evaluation. I was glad to see the issues on NRC redundancy and specificity has been corroborated with new data. Writing and organization have much improved so that the revised manuscript stands as a nice report of the new finding.
The work on NRCs described in this manuscript is quite timely and an important addition to the field in terms of broadening the role NRC helper function in immune signaling upon RLP activation. The presented work will attract broad audience who is well versed in genetics of immunity, disease resistance, immune signaling, who would always like to read PLoS

Genetics articles for inspirational genetics studies. I would agree with other reviewers and the editor that there are some room for improvement in regards to the mechanistic action of NRCs, however, I do not think that detailed mechanistic action is necessary to be included to corroborate genetics experiments. Many times, nice genetic experiments tell much more than we expected to position a factor under a singling pathway, and this piece of work delivers all the necessary points.

We thank the reviewer for their comments and efforts to improve the paper.

I only provide comments that may help clarifying some textual ambiguity.

L165: to me, partial phenotypes are rather more vague than weak phenotypes. Would it mean “partial suppression” or “weak suppression” of Cf-4 mediated cell death upon silencing of NRCs?

We changed this sentence to reflect that here the partial refers to the gene silencing. In the case of Cf4/Avr4 cell death rather than having a complete loss of cell death this can result in weaker cell death (incomplete silencing) or mixed results with loss of cell death in some infiltrations with others not affected (patchy silencing). This can make it hard to interpret the results from these experiments conclusively.

“due also to the partial phenotypes observed using gene silencing constructs targeting the various *N. benthamiana* NRCs” -> “in part due to incomplete silencing of the various N. benthamiana NRCs using previously reported constructs”

L191-192: in L163, the author already mentioned that there is “no” true NRC1 ortholog in N. benthamiana. The way it is descried such as it is NRC3 but “not a N. benthamiana NRC1 homolog as previously implied” is confusing. The argument on "A but not B" applies when A and B are separate entities. Here, the authors rather nicely clarified that previously implied A is A’. Again, here this work identifies that NRC3 in N. benthamiana is the previously suggested NRC1 in N. benthaminan, sorting out confusion by refining the function. I would suggest the authors to make it clear here by revising the sentence (after comma), such as “NRC3 homolog, clearly defining loosely identified a cell death controlling NLR functioning downstream of the Cf-4 RLP”.

We changed this sentence as follows:

“and not a *N. benthamiana* NRC1 homolog as previously implied” -> “thereby clearly defining the contributions of N. benthamiana NRC homologs to pathways downstream of the Cf-4 LRR-RP”"

Have all data underlying the figures and results presented in the manuscript been provided?

Large-scale datasets should be made available via a public repository as described in the *PLOS Genetics*data availability policy, and numerical data that underlies graphs or summary statistics should be provided in spreadsheet form as supporting information.

Reviewer #1: Yes

Reviewer #2: None

Reviewer #3: Yes

PLOS authors have the option to publish the peer review history of their article (what does this mean?). If published, this will include your full peer review and any attached files.

If you choose "no", your identity will remain anonymous but your review may still be made public.

Do you want your identity to be public for this peer review? For information about this choice, including consent withdrawal, please see our Privacy Policy.

Reviewer #1: No

Reviewer #2: No

Reviewer #3: **Yes:** Eunyoung Chae
